# Methanol as an Unlisted Ingredient in Supposedly Alcohol-Based Hand Rub Can Pose Serious Health Risk

**DOI:** 10.3390/ijerph15071440

**Published:** 2018-07-09

**Authors:** Alan P. L. Chan, Thomas Y. K. Chan

**Affiliations:** 1 Division of Clinical Pharmacology and Drug and Poisons Information Bureau, Department of Medicine and Therapeutics, Faculty of Medicine, The Chinese University of Hong Kong, Hong Kong, China; alanchanpl@hotmail.com; 2 Prince of Wales Hospital Poison Treatment Centre, Shatin, New Territories, Hong Kong, China; 3 Asia Pacific Network of Clinical Toxicology Centres, Drug and Poisons Information Bureau, Hong Kong, China

**Keywords:** methanol, poisoning, hand sanitizers, product substitution, risk assessment

## Abstract

Alcohol-based hand rub (hand sanitizer) is heavily used in the community and the healthcare setting to maintain hand hygiene. Methanol must never be used in such a product because oral, pulmonary and/or skin exposures can result in severe systemic toxicity and even deaths. However, sporadic cases of acute poisoning indicate that alcohol-based hand rub with undeclared methanol may be found in the market from time to time. The unexpected presence of methanol poses a serious threat to public health. Unintentional ingestion by young children and inadvertent consumption by older subjects as alcohol (ethanol) substitute can occur. Methanol is more lethal and poisoning often requires antidotal therapy, in addition to supporting therapy and critical care. However, specific therapy may be delayed because the exposure to methanol is initially not suspected. When repeatedly used as a hand rub, skin absorption resulting in chronic toxicity (e.g., visual disturbances) occurs, particularly if methanol induced desquamation and dermatitis are present. Nationwide surveillance systems, regional/international toxicovigilance networks and situational awareness among the healthcare professionals should facilitate the early detection, management and prevention of such poisoning incidents of public health significance.

## 1. Introduction

Hand hygiene products are heavily used in everyday life. In the healthcare setting, most of the cross-infections occur by direct contact via the hands of health workers, and their strict adherence to good hand hygiene is the most effective strategy to stop infections spreading [1]. By reducing cross-transmission, the use of alcohol-based hand rub (hand sanitizer) helps decrease the risk of healthcare associated infections, and hence selection pressure of antibiotics and the emergence of antibiotic resistance [2]. In the community, the combination of hand hygiene and face mask is effective in reducing influenza transmission [3]. In the educational setting, hand hygiene may decrease the risk of respiratory tract infection among children [4]. There are national and WHO guidelines on hand hygiene [5,6]. In general, hands should be washed with soap and water if they are visibly dirty. If hands look clean, alcohol-based hand rub is an effective alternative and, in the healthcare setting, the preferred method for routine hand antisepsis. Hand hygiene products contain an antiseptic to inactivate microorganisms and/or temporarily suppress their growth on the skin [6]. Many preparations are available [6], but alcohol-based hand rub is always preferred because of its greater effectiveness and better compliance rate, while causing less skin irritation and requiring less time for application [1,6].

Alcohol-based hand rub mostly contains ethanol, isopropyl alcohol, *n*-propyl alcohol, or their combinations [2]. When misused, these preparations can become toxic to humans. For example, after accidental or deliberate ingestion, isopropyl alcohol causes more severe central nervous system and respiratory depression than ethanol [7]. Methanol is even more toxic after inhalation, oral or skin exposures [8] and must never be used in hand hygiene products. However, sporadic cases of human poisoning [9] indicate that such hazardous products may be found in the market from time to time. The unexpected presence of methanol poses a serious threat to public health since hand rub is widely used both in the healthcare and community settings.

In this review, the main objective is to describe the serious health risk caused by methanol as an unlisted ingredient in supposedly alcohol-based hand rub. Reports of acute methanol poisoning and chronic toxicity are reviewed.

## 2. Literature Search to Identify Relevant Reports of Methanol Toxicity

To identify published papers in indexed/non-indexed journals, news and other reports, a search of Medicine/Embase (1989 to 29 June 2018), WanFang Data (1989 to June 2018), Google and Google Scholar was performed, using methanol, hand sanitizers, hand hygiene products and alcohol-based hand rub as the keywords. Additional cases, if any, were retrieved from the annual reports of poison control centers [10] and international [11] or national [12,13,14,15] studies of methanol poisoning from the USA [10,12], Europe [11,13,14], Africa [11,15] and Asia [11].

Only reports of acute poisoning (Section 3), following ingestion, inhalation and skin exposure to undeclared methanol in alcohol-based hand rub, were reviewed. Acute methanol poisoning caused by methylated spirits (denatured alcohol) [16] and adulterated alcohol [17] was not included.

Reports of chronic toxicity (Section 4) were also reviewed to characterize the risk and severity of impaired vision and other organ damages, if methanol containing products were inadvertently used as hand rub. Reports of toxic effects when the preparations were used for medicinal purposes [18,19] were excluded because the doses, exposure time and intensity of use were quite different.

## 3. Acute Poisoning Caused by Methanol as an Unlisted Ingredient in Alcohol-Based Hand Rub

The search of biomedical literature databases, Google and Google Scholar altogether identified 3 relevant reports of acute methanol poisoning [9,10,20]. Details about the poisoning incidents and their year of occurrence are summarized in Table 1.

In Canada [9], two Ontario residents had died after ingesting a hand sanitizer. Both bottles were found to contain methanol, an undeclared ingredient, rather than ethyl alcohol, the active ingredient listed on the label. Health Canada issued a warning to consumers and worked with its manufacturer to implement a recall of these products.

In USA [10], a 42-year-old man died after ingesting a hand sanitizer. According to the Annual Report of the American Association of Poison Control Centers, the product contained methanol and ethanol. Methanol was detected in serum (0.31 mmol/L), but the blood sampling time was not stated. No further information was provided.

In Hong Kong [20], a 29-year-old man required intensive care unit care for severe methanol and isopropyl alcohol poisoning after drinking 500 mL of an alcohol-based hand rub. He presented with deep coma (GCS 3/15) and metabolic acidosis (arterial pH 7.28) with an increased osmolar gap (131.5 mOsm/kg). His serum methanol and isopropyl alcohol levels at 4 h post ingestion were 72.0 and 55.9 mmol/L, respectively. Hemodialysis, folinic acid and intravenous ethanol infusion were started. He regained consciousness and was extubated 2 days later. His vision was intact. He was transferred to a psychiatric unit after 9 days of medical treatment. The hand rub was listed to contain isopropyl alcohol, glycerine and triethanolamine, but was found to contain isopropyl alcohol 36%, methanol 22%, and ethanol 3.5%.

## 4. Chronic Toxicity Caused by Methanol Containing Hand Rub

The search of literature databases, Google and Google Scholar had not identified any reports of subacute or chronic toxicity caused by methanol as an unlisted ingredient in alcohol-based hand rub. However, the case series [21] summarized here clearly indicates transdermal absorption of methanol and a very high risk of systemic toxicity among users of alcohol rub containing methanol.

In China [21], a hospital mistakenly purchased industrial (denatured) alcohol for surgeons to disinfect the hands and forearms before surgery. It was used 3–5 sessions per week. There were soon skin dryness and desquamation of the areas exposed to the hand rub. Six months later, 6 surgeons in the same unit developed erythema and rash in the affected areas, with intense itching, especially the fingers and finger web. Four surgeons stopped using this product and recovered spontaneously. The fifth surgeon had further exposures until the skin condition worsened. About 1 month after stopping using the product, he developed mild visual impairment which gradually improved after treatment. The sixth surgeon continued to use the hand rub until blurred vision occurred. Methanol poisoning was suspected. Chemical analysis of the product revealed that its methanol concentration was 3000–5000 times the legal limit. Neither the actual methanol concentration nor its legal limit was stated. There was initial improvement in vision after cessation of use. However, a month later, visual acuity deteriorated to 0.3 and 0.7. Neurological symptoms included lower limb weakness, paroxysmal electric shock sensation in 4 limbs and peripheral neuropathy. Subsequently, further assessments revealed a diagnosis of retrobulbar neuritis and retinitis. Three months later, the visual impairment markedly improved and neurological symptoms gradually improved. Eighteen months later, there was still numbness of left hand fingers.

## 5. Discussion

Alcohol-based hand rub should not contain methanol [6], for obvious reasons. Methanol is very toxic, following oral, pulmonary and/or skin exposures [8]. Severe systemic toxicity and even deaths can occur after occupational or non-occupational exposures [12,13,14,15,17,22,23]. In acute poisoning, the main toxicity of methanol does not manifest until its metabolite formic acid has accumulated to toxic levels, typically 12–24 h after exposure [24]. Apart from severe metabolic acidosis, nausea, vomiting, headache, semi-coma, and ocular toxicity may be seen [8]. Unless timely antidotal therapy is given [25], substantial exposure can result in coma, seizures, death, permanent blindness, and permanent damage to the central nervous system [8,23]. Subacute poisoning has also been reported in workers with inhalation and skin exposures [25]. There are little data on chronic toxicity due to continuous or repeated exposures over a period of time. Chronic inhalation exposure to methanol can result in eye irritation and headache in workers [8]. Rarely, repeated exposures to methanol have been reported to cause visual disturbances and clinical symptoms of multiple sclerosis [21,26].

Albeit very rarely reported, alcohol-based hand rub with methanol as an undeclared ingredient could be found in the market from time to time [9,10,20]. The reasons for its use as the substitute for isopropyl alcohol or ethyl alcohol are not known. The present review highlights the serious threat to public health if such preparations are available to the public (Section 3) and healthcare professionals (Section 4).

Health authorities and the manufacturers repeatedly remind the consumers that alcohol-based hand rub is for external use only. As clearly stated on its product labelling, hand rub should never be swallowed. However, unintentional ingestion by young children is common [27]. In older children, adolescents and adults, hand rub is increasingly used as alcohol (ethanol) substitute rather than for self-harm. [27,28,29]. In fact, consumption of surrogate alcohols (non-beverage alcohols and illegally produced alcohols) is a major public health problem in many parts of the world [30,31]. When taken by mouth, methanol, if ever used as a substitute for isopropyl alcohol or ethanol, in the hand rub will cause a much greater mortality and morbidity. Methanol has a lower lethal dose (~1.2 versus 1–4 and 3.6–6 mL/kg) and poisoning often requires antidotal therapy (fomepizole or ethanol) [24,32], in addition to supporting therapy and critical care [13]. Methanol also causes severe metabolic acidosis and more target organ toxicity (e.g., ocular toxicity) [8]. Specific therapy [24,32] may be inadvertently delayed because the exposure to methanol is initially not suspected.

Obviously, methanol is highly toxic and, if ingested, can be fatal [8]. When used on the skin, it is well known that methanol can cause irritation and inflammation [8]. Dermal exposure resulting in acute poisoning and systemic toxicity remains the main concern [22,23,25]. Exposure assessments in healthy volunteers indicate that the extent and rate of skin absorption of methanol depends on many factors, including its form (vapors, liquid or solution), contact time, dose, concentration, and size of exposure area [33,34,35]. Skin occlusion will enhance the percutaneous absorption of methanol [36], by preventing its evaporation and increasing the stratum corneum hydration and skin temperature. In diseased skin, such as methanol induced desquamation and dermatitis [8,21], both the structure and barrier function become compromised, thus allowing methanol and other chemicals to be absorbed even more readily [37]. The case series described in Section 4 [21] clearly documents skin absorption and a very high risk of systemic toxicity among users of alcohol rub containing methanol.

The poisoning incidents due to undeclared methanol in alcohol-based hand rub are all of major public health significance because of its high intrinsic toxicity, widespread use and easy availability. Nationwide surveillance systems based on, for example, spontaneous reporting and early detection of warning signals by poison control centers [38], will play an important role in the risk management strategies. Toxic threats occurring sporadically in different countries necessitate the establishment of a regional or international toxicovigilance network to detect early warning signals and initiate immediate preventive measures. The present review should enhance the situational awareness among health professionals, who provide timely treatment and sound the alarm.

## 6. Conclusions

Methanol is highly toxic, and severe systemic toxicity and even deaths can occur after oral, pulmonary and/or skin exposures. Therefore, methanol must never be used in alcohol-based hand rub, which mostly contains ethanol, isopropyl alcohol, *n*-propyl alcohol, or their combinations. However, sporadic reports of acute poisoning indicate that hand rub with undeclared methanol may be found in the market from time to time. The unexpected presence of methanol poses a serious threat to public health since hand rub is heavily used and widely available. Unintentional ingestion by young children and inadvertent consumption by older subjects as alcohol (ethanol) substitute can occur. Given the frequent consumption of surrogate alcohols in many parts of the world, methanol, if ever used as a substitute for isopropyl alcohol or ethanol, in the hand rub will cause an even bigger public health problem. Methanol is more lethal and poisoning often requires antidotal therapy, in addition to supporting therapy and critical care. However, specific therapy may be delayed, since the exposure to methanol is initially not suspected. If repeatedly used as a hand rub, skin absorption resulting in chronic toxicity (e.g., visual disturbances) occurs, particularly if methanol induced desquamation and dermatitis are present. Nationwide surveillance systems, regional/international toxicovigilance networks and situational awareness among the healthcare professionals will facilitate the early detection, management and prevention of poisoning incidents of public health significance.

## Figures and Tables

**Table 1:** Reports of acute poisoning caused by methanol in alcohol-based hand rub.

Country (Year)	Details
Canada (2013) [9]	2 deaths after ingesting a hand sanitizer containing methanol (an undeclared ingredient), but ethyl alcohol was the active ingredient actually listed
USA (2014) [10]	M/42 died after ingesting a hand sanitizer containing methanol and ethanol
Hong Kong (2016) [20]	M/29 required hemodialysis and IV ethanol infusion after ingesting a hand sanitizer containing methanol (undeclared) 22%, isopropyl alcohol 36% and ethanol 3.5%
